# Natural Antidiabetic Agents: Insights into Ericaceae-Derived Phenolics and Their Role in Metabolic and Oxidative Modulation in Diabetes

**DOI:** 10.3390/ph18050682

**Published:** 2025-05-04

**Authors:** Mihaela Popescu, Kristina Radivojevic, Diana-Maria Trasca, Renata Maria Varut, Irina Enache, Andrei Osman

**Affiliations:** 1 Department of Endocrinology, University of Medicine and Pharmacy of Craiova, 200349 Craiova, Romania; mihaela.popescu@umfcv.ro; 2 Research Methodology Department, Faculty of Pharmacy, University of Medicine and Pharmacy of Craiova, 200349 Craiova, Romania; kristinaradivojevic03@gmail.com; 3 Department of Internal Medicine, University of Medicine and Pharmacy of Craiova, 200349 Craiova, Romania; 4 Discipline of Anatomy, Department of Anatomy, University of Medicine and Pharmacy, 200349 Craiova, Romania; irina.enache@umfcv.ro (I.E.); andrei.osman@umfcv.ro (A.O.)

**Keywords:** *Ericaceae* plants, antidiabetic effects, diabetes mellitus

## Abstract

Diabetes mellitus (DM) is a chronic disease with a growing prevalence worldwide, leading to severe health complications. Current treatment relies on antidiabetic medications, which may have adverse effects, highlighting the need for alternative approaches. Natural compounds, such as phenolic compounds, have shown promise in glucose modulation. The *Ericaceae* family includes several plants with potential antidiabetic properties. This review examines the pathophysiology of diabetes, chemical composition, and specific *Ericaceae* species that have demonstrated antidiabetic effects. Studies indicate that *Vaccinium* species and other *Ericaceae* plants can lower blood glucose levels and improve insulin sensitivity through mechanisms such as enzyme inhibition. These findings suggest that *Ericaceae* plants may serve as complementary strategies for diabetes management.

## 1. Introduction

Diabetes mellitus has become a fast-growing global health challenge, with over half a billion people affected worldwide [1].

Diabetes is a heterogeneous syndrome identified by defined hyperglycemia which is classified as type 1 diabetes (T1DM), type 2 diabetes (T2DM), specific types of diabetes, and gestational diabetes mellitus [2]. T2DM is a disease of civilization; based on the latest data from the NCD Risk Factor Collaboration, the number of patients was 828 million [3], out of which T2DM accounts for 96% of the patients, and is one of the important non-communicable chronic diseases that seriously threaten human health, without totally clear cognition on pathogenesis [4]. T2DM is a disease outlined by a nonautoimmune heterogeneously progressive loss of adequate islet β cell insulin secretion frequently in the presence of insulin resistance (IR) and metabolic syndrome (MS). It is important to emphasize that type 2 diabetes mellitus (T2DM) is no longer confined to older adults. In recent years, a concerning two- to three-fold increase in the incidence of T2DM has been reported among younger populations, particularly those under 40 years of age [5]. And while diabetes mellitus (DM) is a multifactorial chronic health syndrome affected by several genetic and/or environmental factors [6], T2DM, in particular, is a complex multifactorial polygenetic disease that can be attributed to many risk factors. Some factors include dietary risks, environmental or occupational risks, tobacco use, low physical activity, and alcohol use, all accounting for part of the risk of T2DM [7]. The complications of T2DM are extensive and diverse, including diabetic kidney disease (DKD) developing in 40% of the people with T2DM [8], diabetic retinopathy (DR) where as many as 60% of the people with T2DM were affected after 20 years of disease duration [9], neuropathy which occurs in almost 45% of T2DM patients [10], Metabolic Dysfunction-Associated Steatotic Liver Disease (MASLD) [11], coronary artery disease where mortality due to ischemic heart disease is about two to four times more frequently compared to people free of diabetes [12], and stroke [13] where it was discovered that even prediabetes may also be a cause of higher frequency of stroke [14]. The treatment of T2DM involves pharmacological measures, including oral antidiabetic drugs [15], and some non-pharmacological measures, such as regular physical activity and a healthy diet. Additionally, natural therapeutic alternatives, such as plants, have been sought out due to them containing bioactive compounds with pharmacological properties that intervene in antioxidant action or their mechanisms of action that regulate glucose, among other properties [16,17]. Current pharmacological therapies can mitigate hyperglycemia and slow the progression of diabetic complications, but they do not cure the disease and often carry side effects, underscoring an urgent need for safer and more effective interventions [18]. In response, there is increasing interest in plant-derived antidiabetic agents as complementary or alternative therapeutics. Natural phenolic compounds from medicinal plants have shown promise in improving glycemic control through multi-faceted mechanisms (enhancing insulin sensitivity, inhibiting carbohydrate-hydrolyzing enzymes, and mitigating oxidative stress). In particular, members of the *Ericaceae* family (such as *Vaccinium* berries) are rich in phenolics and have demonstrated notable antidiabetic potential in recent studies [19,20]. The *Ericaceae* family comprises about 4000 species across 126 genera, including prominent genera such as *Calluna*, *Erica*, *Vaccinium, Azelea*, *Rhododendron*, and the *Epacrids* of *Australasia*. The *Ericaceae*, commonly referred to as the heath or heather family, represent a diverse group of flowering plants that predominantly thrive in acidic and nutrient-poor soils. Their remarkable adaptability to such challenging environments has contributed to their widespread distribution across various temperate and subarctic regions [21]. The *Ericaceae* family has an extensive and diverse range of compounds such as phenolic compounds, pectin, vitamins, sugars [22,23], and anthocyanins (ANTs) [24]. Some of the compounds can be exclusively found in this family, such as grayanane diterpenes [25] which have analgesic [26,27], anti-inflammatory [28], antifeedant [29], and protein tyrosine phosphatase 1B (PTP1B) [30] inhibitory activities. Additionally, studies have found that extracts with phenolic compounds showed powerful α-glucosidase inhibitory activity and it is even more efficacious than the marketed drug acarbose. Moreover, the glucosidase inhibitory activities of *Rhododendron arboreum* were found to be many-fold higher than those of acarbose [31].

The decision to focus this review on the *Ericaceae* family is motivated by the unique combination of phytochemical richness and bioactivity exhibited by its members. Ericaceous plants stand out for their exceptional abundance and diversity of phenolic compounds—including anthocyanins, flavonoids, and tannins—which contribute to potent antioxidant effects and multifaceted antidiabetic activities documented in the literature [32,33]. Moreover, many *Ericaceae* species have a long history of ethnomedicinal use in glycemic control; for example, bilberry (*Vaccinium myrtillus*) leaf infusions were widely used as a traditional remedy for diabetes before the advent of insulin therapy [34]. This family also produces unique secondary metabolites, such as grayanane diterpenes found almost exclusively in *Ericaceae*, which display bioactivities relevant to diabetes (notably the inhibition of protein tyrosine phosphatase 1B, a negative regulator of insulin signaling). These attributes, together with robust evidence from both in vitro experiments and in vivo studies, including preliminary clinical trials showing improved glycemic indices with *Ericaceae*-derived interventions, provide a compelling rationale for prioritizing the *Ericaceae* family over other plant families in the search for natural antidiabetic agents [35]. Accordingly, the objective of this review is to comprehensively examine *Ericaceae*-derived phenolics and their roles in modulating metabolic and oxidative pathways, highlighting the links between phytochemical profiles and therapeutic mechanisms in diabetes management.

## 2. Pathophysiology of Diabetes

T1DM and T2DM are the most common subtypes of DM. Type 1 occurs mainly in children or adolescents [36], while type 2 usually affects middle-aged and elderly adults who have persistent hyperglycemia, mainly due to genetic variants, inappropriate lifestyle, and dietary habits. The pathogenesis of these two types is meaningfully different, so each type is characterized by a distinct etiology, pathophysiology, presentation, and treatment [36,37]. T1DM is characterized by elevated blood glucose levels (hyperglycemia) caused by deficient insulin production due to the destruction of the β-cells of the pancreatic islets of Langerhans, predominantly because of autoimmune inflammation [36]. The traditional understanding posits that autoreactive T cells mistakenly target and destroy healthy pancreatic β-cells, leading to insulin deficiency and consequent hyperglycemia [reference]. This loss of insulin activity not only impairs glucose uptake and metabolism in peripheral tissues such as muscle and adipose tissue, but also promotes excessive hepatic glucose production through enhanced glycogenolysis and gluconeogenesis, processes that are further stimulated by elevated glucagon levels [38]. During the early stages of type 1 diabetes mellitus (T1DM), the seroconversion of islet-specific autoantibodies—targeting insulin, glutamate decarboxylase, insulinoma-associated antigen 2, or zinc transporter 8—represents the earliest notable indicator of autoimmune activity. The simultaneous presence of multiple autoantibodies in the serum remains the most reliable predictor for both the loss of immune tolerance and the eventual clinical onset of T1DM, even though the precise role of these autoantibodies in β-cell destruction is not yet fully understood [39]. As the disease progresses, immune cells infiltrate the pancreatic islets, generating a pro-inflammatory microenvironment characteristic of insulitis. This inflammatory state not only facilitates further β-cell injury but also enhances the presentation of islet antigens via HLA class I molecules, thereby perpetuating autoimmune responses and accelerating the development of T1DM [40]. Type 2 diabetes mellitus (T2DM) is a complex metabolic disorder in which insulin resistance and impaired insulin secretion are primarily driven by the patient’s overweight or obesity status. Epidemiological data indicates that approximately 86% of individuals with T2DM are overweight, underscoring the critical link between excess adiposity and the pathophysiology of the disease. The chronic low-grade inflammation, altered adipokine secretion, and ectopic lipid accumulation associated with obesity collectively contribute to the deterioration of insulin sensitivity and β-cell function [41]. Recent evidence suggests that β-cell dysfunction in type 2 diabetes mellitus (T2DM) arises from a complex interplay between environmental factors and various molecular pathways involved in cellular homeostasis [42]. In states of nutritional excess, such as those observed in obesity, the simultaneous presence of hyperglycemia and hyperlipidemia fosters insulin resistance (IR) and a chronic inflammatory milieu. Under these conditions, β-cells—depending on their genetic susceptibility—are exposed to multiple toxic insults, including inflammatory, endoplasmic reticulum (ER), metabolic, oxidative, and amyloid stresses, ultimately jeopardizing islet integrity and function [43]. An overload of free fatty acids (FFAs) and persistent hyperglycemia promotes ER stress by activating pro-apoptotic branches of the unfolded protein response (UPR) pathways, thereby contributing to β-cell dysfunction [44]. Lipotoxicity, glucotoxicity, and glucolipotoxicity—hallmarks of obesity—further exacerbate metabolic and oxidative stress within β-cells, accelerating their deterioration [42]. Mechanistically, elevated levels of saturated FFAs can impair ER homeostasis by inhibiting the sarco/endoplasmic reticulum Ca^2^⁺-ATPase (SERCA), activating inositol 1,4,5-triphosphate (IP3) receptors, and disrupting calcium mobilization within the ER. Moreover, chronic hyperglycemia increases proinsulin and islet amyloid polypeptide (IAPP) synthesis in β-cells, leading to the accumulation of misfolded proteins and enhanced generation of reactive oxygen species (ROS) via oxidative protein folding processes [44]. These disturbances further impair ER calcium handling, amplify pro-apoptotic signaling, degrade proinsulin mRNA, and promote interleukin-1β (IL-1β) release, recruiting macrophages and intensifying local islet inflammation [42].

Maintaining proper insulin secretion is vital to meet the body’s metabolic demands, necessitating the preservation of islet architecture and coordinated cell-to-cell communication. The disruption of islet integrity, as driven by the aforementioned stressors, impairs the synchronized secretion of insulin and glucagon, contributing to worsening hyperglycemia. Ultimately, defects in insulin precursor synthesis, insulin production, or secretion mechanisms form the core of β-cell failure, establishing a pathological foundation for T2DM [45].

## 3. Chemical Composition of Ericaceae Plants with Antidiabetic Potential

The secondary metabolites of the plants protect them from various microbial attacks and have potent medicinal properties. Based on their chemical structures, secondary metabolites are categorized into several classes, such as phenolics, alkaloids, saponins, terpenes, and lipids [46]. Flavonoid content in various plants from the *Ericaceae* family with antidiabetic effects is shown in Table 1. Table 2 summarizes key phenolic compounds identified in the studied Ericaceae species, providing their IUPAC names, molecular formulas, and corresponding 2D structures. The compounds listed include flavonoids such as quercetin, kaempferol, and myricetin, as well as anthocyanidins such as cyanidin, delphinidin, malvidin, petunidin, and pelargonidin. These bioactive molecules are known for their antioxidant, anti-inflammatory, and potential antidiabetic properties, and their structural diversity underpins their wide range of biological activities. Among these compounds, the phenolic group attracts considerable interest as the most promising secondary metabolite for the treatment of several diseases, including diabetes (Table 3). Recent research has identified several phenolic compounds in *Ericaceae* plants that exert antidiabetic effects through multi-enzymatic inhibition and the modulation of key metabolic pathways. Among these, anthocyanins such as delphinidin, cyanidin, and malvidin—commonly found in *Vaccinium* species—demonstrate strong inhibitory effects on α-glucosidase and α-amylase, thereby slowing carbohydrate digestion and blunting postprandial glucose spikes. Flavonols like quercetin and myricetin, present in *Arbutus unedo*, *Rhododendron arboreum*, and *Gaultheria trichophylla*, also inhibit aldose reductase, a key enzyme in the polyol pathway implicated in diabetic complications, as well as protein tyrosine phosphatase 1B (PTP1B), a negative regulator of insulin signaling [47]. Catechins and proanthocyanidins, abundant in *Gaultheria* and *Vaccinium vitis-idaea*, inhibit dipeptidyl peptidase-IV (DPP-IV), an enzyme that degrades incretin hormones like GLP-1, thereby prolonging insulin secretion and enhancing glycemic control. Moreover, arbutin, the major phenolic glycoside in *Arctostaphylos uva-ursi*, has been shown to inhibit glucose-6-phosphatase, thereby suppressing hepatic gluconeogenesis and contributing to lower fasting blood glucose levels. In addition to direct enzyme inhibition, several *Ericaceae*-derived flavonoids activate AMP-activated protein kinase (AMPK) and upregulate GLUT4 expression, facilitating glucose uptake in skeletal muscle and adipose tissues [48]. This integrative mode of action underscores the therapeutic potential of *Ericaceae* phenolics, which not only inhibit carbohydrate-digesting enzymes but also modulate intracellular signaling and metabolic enzyme pathways involved in glucose homeostasis. Interestingly, many edible *Ericaceae* fruits accumulate substantial tannins (condensed proanthocyanidins and hydrolyzable gallo-/ellagitannins), a class under-reported in prior reviews. For example, Vaccinium berries (blueberry, bilberry, cranberry, and lingonberry) are noted as “rich in proanthocyanidins”, and bilberry, in particular, contains both A- and B-type PA oligomers; similarly, the Mediterranean strawberry tree (Arbutus unedo) fruits yield galloyl (gallotannin) and ellagitannin derivatives. Such tannins likely contribute to antidiabetic effects via multiple mechanisms. In vitro, removing tannins greatly weakens the berry-extract inhibition of α-amylase, and isolated A/B-type proanthocyanidin fractions (e.g., from lingonberry) block α-amylase and α-glucosidase while enhancing hepatic glucose uptake. Concurrently, condensed tannins are potent antioxidants/anti-inflammatories: Vaccinium PACs suppress oxidative stress and improve insulin sensitivity, and dietary tannins (e.g., tannic acid or red-wine tannins) markedly blunt postprandial glycemic spikes in humans, akin to acarbose [49]. Chronic hyperglycemia in diabetes drives the excess production of reactive oxygen species (ROS), creating an oxidative environment that damages pancreatic β-cells and impairs insulin signaling. This stress directly oxidizes cellular proteins and lipids and activates stress-sensitive kinases (JNK or NF-κB) that inhibit the insulin receptor signaling cascade, thereby promoting insulin resistance. Because β-cells have intrinsically low antioxidant defenses (e.g., low levels of superoxide dismutase, catalase, and glutathione peroxidase), this ROS-mediated injury particularly impairs insulin secretion [50]. By contrast, antioxidant-rich extracts from *Ericaceae* plants (for example, anthocyanin- and polyphenol-rich berries) can scavenge excess ROS and reduce inflammation; indeed, Vaccinium extracts have been shown to lower oxidative stress, inflammation, and blood glucose in diabetic models. In this way, the antioxidative properties of these plants help restore redox balance and preserve insulin sensitivity, providing a mechanistic rationale for their dual antidiabetic and antioxidant effects. Out of all phenolic compounds, flavonoids are the most prevalent and well-studied class of polyphenols. Studies have shown that flavonoids possess powerful antidiabetic and antioxidant activities (Table 4) [51,52,53].

## 4. *Ericaceae* Phenolic Compounds and Enzyme Inhibition in Diabetes

**Figure 1:**
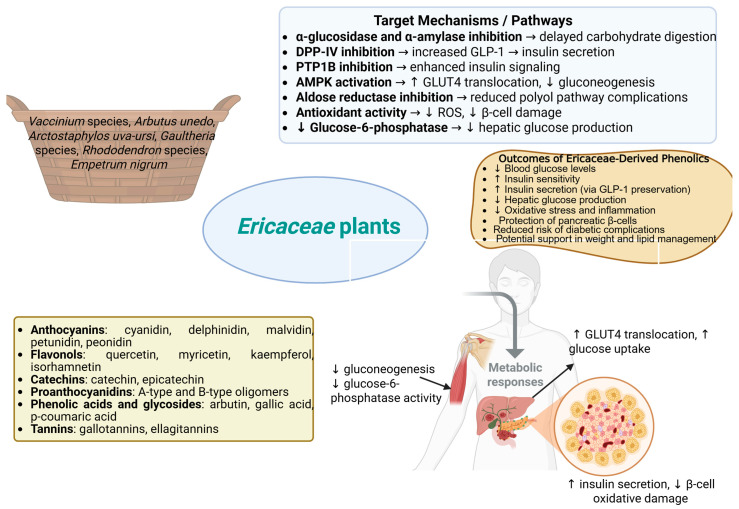
Graphical summary of Ericaceae-derived phenolic compounds, their molecular targets, and physiological outcomes in diabetes management. Created with BioRender.com: https://app.biorender.com/illustrations/6812a4fbba4c52be0bd59244 (accessed on 14 March 2025).

Figure 1 highlights the key Ericaceae plant sources, major classes of bioactive phenolics, and their mechanistic actions on metabolic pathways such as glucose uptake, insulin secretion, and hepatic gluconeogenesis, ultimately contributing to improved glycemic control and protection against diabetic complications.

### 4.1. Inhibition of Digestive Enzymes (α-Glucosidase and α-Amylase)

Phenolic compounds from *Ericaceae* (e.g., blueberries, bilberries, and bearberry) can moderate postprandial glycemia by inhibiting α-glucosidase and α-amylase, the key enzymes that digest dietary carbohydrates. Anthocyanins are particularly effective: a purified anthocyanin extract showed an IC_50_ ~0.71 mg/mL against α-glucosidase, markedly more potent than acarbose (IC_50_ ~8.8 mg/mL). The same extract inhibited α-amylase with IC_50_ ~1.14 mg/mL (comparable to acarbose, ~1.0 mg/mL) [92]. Kinetic analyses indicate a mixed or uncompetitive inhibition mode for these anthocyanins, suggesting they bind to the enzyme-substrate complex or allosteric sites to impede carbohydrate breakdown. In molecular docking studies, flavonols like myricetin (commonly found in *Ericaceae* berries) can anchor into the α-amylase active site via multiple hydrogen bonds (to catalytic residues Asp197, Asp300, Asp356, etc.) and π–π stacking with Trp59 [93], directly blocking the starch-binding cleft. Likewise, the phenolic glycoside arbutin (abundant in bearberry *Arctostaphylos uva-ursi*) exhibits direct α-amylase and α-glucosidase inhibition in vitro. Although arbutin alone is slightly less potent than whole plant extracts (which contain synergistic flavonoids and tannins), it contributes to the overall enzyme-inhibitory activity of *Ericaceae* preparations. Through these mechanisms, berry phenolics slow the release of glucose from complex carbs, flattening postprandial glucose spikes. In vivo, this translates to improved glycemic control and insulin economy, analogous to pharmaceutical α-glucosidase inhibitors but potentially with fewer gastrointestinal side effects [94].

### 4.2. DPP-IV Inhibition and Incretin Enhancement

*Ericaceae* phenolics also target dipeptidyl peptidase-IV (DPP-IV), an enzyme that degrades the incretin hormones GLP-1 and GIP. The inhibition of DPP-IV prolongs incretin action, thereby enhancing glucose-stimulated insulin secretion and lowering blood glucose. Several berry-derived polyphenols act as natural DPP-IV inhibitors. Notably, anthocyanins and flavonols can achieve low-micromolar or sub-micromolar potency. For example, cyanidin-3-glucoside (a major anthocyanin in bilberry) inhibits DPP-IV with an IC_50_ ≈ 0.4 μM, and cyanidin aglycone around 1.4 μM. Quercetin, a flavonol present in blueberries, has an IC₅₀ in the low micromolar range (~2–3 μM). These values approach or even surpass the potency of a known peptidic inhibitor (Diprotin A, IC_50_ ~4.2 μM). Mechanistically, docking studies reveal that polyphenols like cyanidin-3-glucoside and quercetin fit into the DPP-IV active site, interacting with key subsites S2/S3 via multiple hydrogen bonds and π-interactions. For instance, quercetin’s hydroxyl groups form hydrogen bonds with residues such as Arg356 and Arg358 in the catalytic pocket, while its aromatic rings stack against hydrophobic pocket residues [95]. This mimics how synthetic inhibitors bind DPP-IV, effectively blocking incretin degradation. Consistent with these molecular insights, berry extracts rich in these phenolics have shown DPP-IV inhibitory activity in cell-free assays and even in animal models. One study noted that grape seed procyanidins (structurally similar to berry proanthocyanidins) reduced intestinal DPP-IV activity by ~34% after a single dose, suggesting that dietary polyphenols can acutely elevate circulating GLP-1. By preserving endogenous incretins, *Ericaceae* phenolics help sustain insulinotropic signaling, improve meal-time insulin release, and attenuate postprandial hyperglycemia [96].

### 4.3. Aldose Reductase Inhibition and Polyol Pathway Protection

Chronic hyperglycemia in diabetes drives the polyol pathway, where excess glucose is reduced to sorbitol by the enzyme aldose reductase. Sorbitol accumulation causes osmotic and oxidative stress, contributing to complications like neuropathy, retinopathy, and cataracts. Phenolic compounds from *Ericaceae* can mitigate this by inhibiting aldose reductase (AR). Flavonols are particularly potent AR inhibitors: quercetin and its glycosides (quercitrin and myricitrin) were shown to be significantly more potent than classic AR inhibitor drugs in lens assays. These flavonols exhibited noncompetitive inhibition kinetics against AR, implying they bind to a site distinct from the glucose/NADPH active site (possibly an enzyme-cofactor or enzyme-product complex), thereby modulating enzyme activity without directly competing with the substrate. In functional terms, quercitrin was able to block sorbitol accumulation in isolated rat lenses exposed to high glucose, confirming that flavonoid AR inhibitors work in intact tissues [97]. Anthocyanins and catechins in *Ericaceae* likely contribute to AR inhibition as well, as many polyphenols share a structural capacity to bind AR’s active pocket or adjacent sites. By reducing flux through the polyol pathway, these compounds help prevent the intracellular sorbitol buildup that underlies diabetic cataract formation and nerve damage. This protective mechanism has been borne out in vivo: quercetin supplementation in diabetic rats lowered retinal sorbitol levels and improved oxidative stress markers, highlighting the relevance of AR inhibition in complication prevention. Thus, *Ericaceae* phenolics not only control blood sugar levels but also directly guard tissues against hyperglycemic injury by targeting aldose reductase [98].

### 4.4. PTP1B Inhibition and Insulin Signaling Enhancement

Beyond effects on digestive enzymes and hormones, *Ericaceae* phenolics improve insulin sensitivity at the cellular level by inhibiting protein tyrosine phosphatase 1B (PTP1B). PTP1B is a negative regulator of insulin signaling—it dephosphorylates the insulin receptor (IR) and insulin receptor substrates, attenuating the PI3K/Akt pathway. In obesity and type 2 diabetes, PTP1B is often overexpressed, contributing to insulin resistance. Remarkably, anthocyanin-rich berry extracts have demonstrated potent PTP1B inhibition. For example, blueberries and bilberries (*Vaccinium* spp.) yielded anthocyanin fractions with IC_50_ ~3 μg/mL against PTP1B. Among individual compounds, cyanidin-3-O-glucoside stands out: docking studies show it can occupy both the active site and an adjacent allosteric site of PTP1B, with a binding energy of around –7.8 kcal/mol [99]. By engaging the enzyme in this bidentate manner, cyanidin-3-glucoside effectively blocks PTP1B activity. Flavonols present in *Ericaceae* (quercetin and myricetin) and even flavan-3-ols (catechins) may also contribute to PTP1B inhibition [100], as these classes have reported PTP1B-inhibitory activity in other plant systems. The downstream consequence of PTP1B inhibition is an enhancement of insulin signaling. With PTP1B activity restrained, the insulin receptor stays phosphorylated for longer upon insulin binding, leading to the amplified recruitment of PI3K and activation of Akt. This has been observed as increased Akt phosphorylation and GLUT4 translocation in muscle and adipose tissues treated with berry polyphenols. In one study, myricetin administration to insulin-resistant rats elevated the phosphorylation of IR, IRS-1, and Akt in skeletal muscle, thereby promoting GLUT4 mobilization to the membrane. Notably, that study linked myricetin’s action to an upstream rise in β-endorphin (acting on opioid receptors) revealing a complementary mechanism by which a flavonol can alleviate insulin resistance. Overall, by inhibiting PTP1B, *Ericaceae* phenolics release a brake on the insulin signaling cascade, restoring insulin sensitivity and improving glucose uptake in peripheral tissues [101].

### 4.5. Activation of AMPK and Modulation of Glucose Metabolism

In addition to direct enzyme inhibition, *Ericaceae*-derived phenolics influence cellular energy-sensing pathways, most importantly the AMP-activated protein kinase (AMPK). AMPK is a central metabolic regulator that, when activated, stimulates glucose uptake and fatty acid oxidation while suppressing gluconeogenesis. Polyphenols like quercetin and anthocyanins are known to activate AMPK in liver and muscle cells. Quercetin has been shown to increase the Thr172-phosphorylation of AMPK in insulin-resistant hepatocytes and myotubes, in turn downregulating gluconeogenic enzymes including glucose-6-phosphatase and PEPCK. This leads to reduced hepatic glucose output and lower fasting glycemia. Concordantly, quercetin-treated diabetic animals exhibit decreased blood glucose along with suppressed hepatic G6Pase activity and gene expression [102]. Anthocyanins can likewise engage AMPK-dependent pathways: black rice anthocyanin extracts, as a model, stimulated AMPK (and p38 MAPK) in skeletal muscle cells, increasing GLUT4 translocation and glucose uptake independently of insulin. Interestingly, the same extracts also enhanced insulin-dependent signaling (IRS-1 and PI3K/Akt), indicating that anthocyanins act on multiple nodes to facilitate glucose disposal. In the muscle and adipose tissue of diabetic models, anthocyanin-rich diets have been found to elevate GLUT4 abundance and incorporation into the plasma membrane, an effect attributed to the upregulation of AMPK activity and restoration of insulin sensitivity in those tissues [103]. Besides improving glycemic control, AMPK activation by these compounds has favorable effects on lipid metabolism (e.g., inhibition of lipogenesis and stimulation of fatty-acid oxidation) and on GLUT4 gene expression in muscle. It is postulated that polyphenols may activate AMPK by causing mild cellular stress or mitochondrial inhibition (somewhat analogous to metformin’s mechanism), thereby increasing the AMP/ATP ratio. For example, catechin-type polyphenols can depolarize mitochondria and activate AMPK in the liver, contributing to decreased gluconeogenic output. Through AMPK, *Ericaceae* phenolics orchestrate a shift from glucose production to glucose utilization. The net result is improved whole-body glucose homeostasis: enhanced muscle glucose uptake (via more GLUT4 on cell surfaces) and diminished liver glucose release. This synergizes with their insulin-sensitizing effects (via PTP1B inhibition) to overcome insulin resistance [104].

## 5. In Vitro and In Vivo Antidiabetic Studies for Vaccinium Species

Belonging to the *Ericaceae* family (notably the *Rhododendron* genus), the *Vaccinium* genus consists of a morphologically diverse group of approximately 4250 species (33 types), divided into nine subfamilies and 125 genera, with prevalence mostly across Europe, Southeast and Central Africa, Asia, and North and Central America [90,91]. The wild species of this genus that are prevalent in Europe include *V. myrtillus* L. (bilberry), *V. vitis-idaea* L. (lingonberry), *V. oxycoccus* L. (cranberry), and *V. uliginosum* L. (bog bilberry) [105]. The most widely cultivated species within the Ericaceae family are found in the subfamily *Vaccinioideae*, which includes economically and nutritionally important fruits such as cranberry, blueberry, huckleberry, and bilberry [106]. According to multiple studies, the principal health benefits associated with these fruits are largely attributed to their potent antioxidant, antimicrobial, and detoxifying effects on the human body [reference]. These biological activities underpin their growing recognition as functional foods with the potential to support overall health and prevent various chronic diseases [107]. Moreover, it is widely recognized that these plants, especially those rich in polyphenolic compounds such as anthocyanins, possess the ability to inhibit cancer cell proliferation and promote apoptosis within malignant cells [reference]. This dual action highlights their promising role as natural agents in cancer prevention and adjunctive therapy [108,109,110]. Given their benefits and the widespread presence of this genus, it is predictable that numerous studies have explored their potential in diabetes mellitus (Table 5).

### 5.1. Bilberry

**Figure 2:**
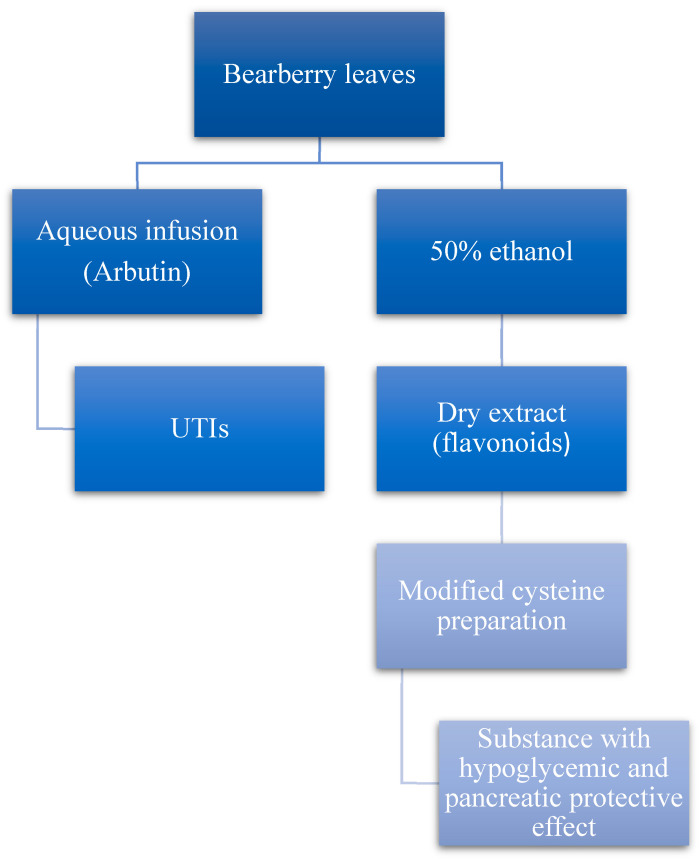
Therapeutic applications of bearberry (*Arctostaphylos uva-ursi*) leaf extracts. The diagram illustrates both the traditional and emerging uses of bearberry. Aqueous infusions containing arbutin are used for urinary tract infections, while ethanol extracts rich in flavonoids, formulated with cysteine, have shown hypoglycemic and pancreatic protective effects in experimental settings.

Bilberry (*Vaccinium myrtillus*) is a perennial, low-growing shrub that can reach a height of 35–60 cm. It grows in acidic soils [121], organic forest soils, mountainous mineral heaths, and old peat bogs in central and northern parts of Europe [122]. Bilberry is commonly known as the European blueberry or whortleberry [121]. An uncommon albino form is characterized by greenish-white fruits, an appearance caused by the suppression of genes involved in anthocyanin synthesis [123]. The fruits are used, often in combination with other ingredients, to produce syrups, pies, tarts, and beverages. The leaves are generally used to prepare decoctions [121,124]. The growing demand for a berry-rich diet has led to the increased consumption and cultivation of two Vaccinium species: *V. myrtillus* (wild bilberries) and *V. corymbosum* (cultivated blueberries) [125]. Numerous studies [126] have shown that the consumption of bilberry fruit improves cellular function and glycemic control in diabetic patients. It has been shown that bilberry consumption increases hippuric acid levels in fasting serum, and over time, this can enhance glucose and insulin metabolism. Bilberry extracts also appear to improve eye microcirculation and reduce intraocular pressure [127]. Bilberry is used as an adjuvant therapy (alongside a proper diet) in the early stages of type 2 diabetes [128]. In vitro studies have demonstrated that V. myrtillus leaf extracts inhibit the activity of α-glucosidase and α-amylase [129], helping to prevent hyperglycemia by enhancing pancreatic beta cell function. A reduction in body weight was also observed [130]. Given these health benefits, several studies have explored the antidiabetic potential of bilberry fruits and leaves in T2DM (Figure 2). Two clinical studies from China tested bilberry extract in patients with T2DM, measuring HbA1c as a primary outcome. One study reported a significant 8.5% reduction in fasting blood glucose levels [131], while the other showed a 4.7% decrease in HbA1c in subjects with prediabetes and T2DM [85]. However, a recent study reported the negligible effects of bilberry and grape seed extract on glucose and cholesterol metabolism, although a significant reduction in blood pressure was observed. The limited outcome may be attributed to the small sample size (14 participants) [132]. Another study found a correlation between myricetin content and the degree of α-amylase inhibition, further confirming that bilberry polyphenols play a role in this enzymatic suppression [133]. In vivo experiments investigating non-acylated anthocyanin extracts from bilberries in diabetic rats revealed modulatory or even restorative effects on abnormal urinary metabolite profiles [134]. Additionally, a study developed fast-dissolving films using β-glucan and bilberry juice, capitalizing on the bioactive properties of both components for use in packaging dry powdered antidiabetic medications [135].

The breadth of research on bilberry’s applications in diabetes supports its promising potential as a complementary strategy in diabetes management.

### 5.2. Cranberry (V. sect. Oxycoccus)

Cranberry is a diploid fruit [136], classified as a woody perennial plant characterized by the production of vertical stems [137]. It is taxonomically divided into four main species: *Vaccinium erythrocarpum* (southern mountain cranberry), *V. macrocarpon* (large cranberry, American cranberry, or bearberry), *V. microcarpum* (small cranberry), and *V. oxycoccos* (common or northern cranberry) [138]. Among these, *V. macrocarpon* (American cranberry) and *V. oxycoccos* (European cranberry) are the most widely cultivated and studied species. Cranberries are particularly valued for their rich content of bioactive compounds, including anthocyanins; flavonols; flavan-3-ols (catechins); proanthocyanidins (PACs); benzoic and phenolic acids; nonflavonoid polyphenols such as phloridzin; and terpenes and sterols [139]. *V. macrocarpon* stands out both as a traditional medicinal agent and a nutritional food source, largely due to its complex phytochemical profile. Among the more than 150 identified compounds, flavonoids represent the dominant class, encompassing 13 anthocyanins, 16 flavonols, and 26 phenolic acids and benzoates. This rich composition underpins the diverse therapeutic properties attributed to American cranberries [140]. For cranberry fruit, Cermak et al. [141] showed that quercetin glucosides inhibit glucose uptake into the vesicles of the brush membrane of the pig intestine. Strobel et al. [142] demonstrated that the myricetin present in fruit can inhibit glucose assimilation via the type 4 glucose transporter by rat adipocytes. Schell et al. [119] found that a diet enriched with low-calorie cranberries had a significant effect on improving postprandial glucose levels, and Rocha et al. [143], that the daily consumption (240 mL) of cranberry juice improved glucose control in patients with type 2 diabetes. As for the application of cranberry in T2DM, studies included both glucose-lowering (Figure 2) and complication-beneficial effects research. In several studies investigating the glucose-lowering effects of cranberry supplementation, the Homeostatic Model Assessment of Insulin Resistance (HOMA-IR) was employed as a practical tool to evaluate insulin resistance, a key factor associated with the risk of CVD and T2DM [144]. HOMA-IR is a calculated index based on fasting insulin and fasting blood glucose (FBG) levels [144]. Three clinical trials reported a significant reduction in HOMA-IR values among participants receiving cranberry interventions compared to those in placebo groups, although other metabolic parameters did not show notable differences [121,145,146]. Two studies conducted in Iran further demonstrated that cranberry supplementation could positively influence insulin levels [145,146]. The mechanisms underlying glycemic regulation were hypothesized to be largely attributed to the polyphenolic content of cranberries, a hypothesis supported by numerous animal studies [147,148,149]. Additionally, an acute clinical study on obese individuals with T2DM revealed that postprandial blood glucose levels were significantly lower in participants consuming cranberries compared to controls [150]. The form of cranberry used in interventions appeared to be an important determinant of metabolic outcomes. Studies administering dried cranberry preparations—such as capsules, powders, or tablets—reported more significant improvements in insulin levels compared to those using cranberry juice. Five studies utilized dried forms, with Hormoznejad et al. and Shirazi et al. both noting substantial reductions in insulin concentrations and HOMA-IR scores within the intervention groups [145,146]. Furthermore, Flanagan et al. identified a beneficial effect of cranberry consumption on lipid profiles, suggesting broader cardiometabolic advantages [151]. Arginine-loaded extracts from American cranberry leaves have also been investigated for their potential to improve insulin resistance (IR). The experimental results in rats demonstrated a reduction in body weight, accompanied by decreased triglyceride (TG) accumulation in the liver. Additionally, the serum levels of high-density lipoprotein cholesterol (Ch-HDL) were found to be negatively correlated with HOMA-IR values, suggesting an improvement in insulin sensitivity [152]. Other studies researched the efficacy of cranberry in the complications of T2DM. For wound-healing potential among individuals with diabetes, hydrogel containing cranberry extract and graphene-oxide was used, where animals receiving electroactive and photothermal treatment showed a notably higher percentage of wound healing than the other groups [153]. Another double-blind, placebo-controlled clinical study showed a statistically significant reduction in urinary tract infection (UTI) episodes in the supplemented group compared to placebo administration in T2DM patients [154], where the occurrence is higher. As cranberry consumption lowers the risk of type 2 diabetes [119], the potential for cranberries in the treatment and in the prevention of complications is shown in a high number of studies.

### 5.3. Blueberry

Blueberries (*Vaccinium* spp.) are small, spherical fruits native to North America [155]. Notably, the consumption of blueberry juice has been associated with improvements in memory function among older adults experiencing early signs of cognitive decline. Given their versatile phytochemical composition, blueberries have garnered considerable research interest in the context of T2DM.

With respect to glycemic control, 21 studies have evaluated the effects of blueberry consumption on fasting blood glucose and/or HbA1c levels [156,157,158,159]. The majority of these investigations found no significant differences between intervention and control groups. However, several animal studies reported a reduction in blood glucose levels following dietary supplementation with blueberries [158,160], and one study documented a significant decrease in HbA1c levels in rats treated with blueberries compared to controls [161]. Regarding insulin dynamics, four studies demonstrated that blueberry supplementation led to lower insulin levels in mice and rats relative to control groups, suggesting a potential role for blueberries in improving insulin sensitivity [157,162,163,164]. In contrast, other studies observed similar insulin levels between experimental groups [156,158,161]. Moreover, six studies reported a decrease in IR in animals from the fruit group [152,153,154,156,157,158], while two other studies did not observe any reduction in IR in the treated group (vs. controls) [160,161]. Interestingly, Brader et al. [155] reported that rats fed with a blueberry-enriched diet exhibited decreased hepatic expression of glucose transporter 2 (Glut2) and insulin receptor substrate 1 (Irs1), alongside increased expression of Glut4 in adipose tissue compared to controls. Similarly, Seymour et al. [162] observed the upregulation of both Glut4 and Irs1 in adipose tissue and skeletal muscle, as well as elevated expression of uncoupling protein 3 (Ucp3) in the skeletal muscle of blueberry-fed rats. In another study. Liu et al. [156] suggested that blueberries may improve glucose tolerance by enhancing pancreatic β-cell survival and reducing the expression of pro-inflammatory cytokines and oxidative stress markers. Additional mechanisms proposed for the blueberry-induced improvements in glucose metabolism include the upregulation of Glut2 and Glut4 expression [162,165], activation of peroxisome proliferator-activated receptor (PPAR) pathways and AMP-activated protein kinases (AMPKs), and downregulation of retinol-binding protein 4 (RBP4) expression [166]. Furthermore, the modulation of the gut microbiota following blueberry consumption may also contribute to enhanced glucose tolerance [167]. The potential of blueberries in diabetes prevention has been investigated in epidemiological studies, where a higher habitual intake of blueberries was associated with a lower risk of developing type 2 diabetes [167,168,169]. In particular, a prospective longitudinal cohort study reported that consuming two or more servings of blueberries per week was linked to a significantly reduced risk of T2DM compared to infrequent consumption (less than one serving per month) [147]. Clinical trials have provided mixed results regarding the impact of blueberries on insulin sensitivity. In a randomized controlled trial (RCT) involving adults with obesity and prediabetes, daily blueberry intake (equivalent to 300 g fresh blueberries, providing 668 mg of anthocyanins) over six weeks significantly improved insulin sensitivity [170]. However, other studies found no significant improvements in insulin sensitivity after intervention periods of 6 and 24 weeks among individuals with prediabetes [171,172]. Moreover, a 12-week blueberry supplementation study in individuals with prediabetes and subjective cognitive decline reported lower fasting insulin levels, although no significant changes were observed in fasting blood glucose or HOMA-IR scores [173]. An acute clinical trial also demonstrated that consuming blueberries (equivalent to 1 cup or 150 g fresh blueberries, providing 364 mg of anthocyanins) significantly reduced postprandial blood glucose and insulin concentrations over a 24 h period following an energy-dense, high-fat/high-sugar meal [174].

### 5.4. Lingonberry (Vaccinium vitis-idaea L.)

Lingonberry (*Vaccinium vitis-idaea* L.) is a small red berry that grows wild across the forests of Northern countries, Central Europe, Russia, and Canada [175]. Although the majority of lingonberries are harvested from wild populations, limited cultivation efforts exist, and plant breeding programs for this species remain in their early stages [176]. Closely related to the cranberry (*Vaccinium oxycoccos*), lingonberries are comparatively less well known and less commercially popular. The fruits of *V. vitis-idaea* are rich in essential nutrients, including a variety of vitamins, polysaccharides, dietary fiber, and minerals. They also contain an array of bioactive compounds such as anthocyanins, proanthocyanidins, flavonols, phenolic acids, simple phenolics, phytosterols [177], hydroxycinnamic acids, triterpenoids, and flavonoids, which collectively contribute to their recognized health benefits [178]. Lingonberries exhibit strong anti-inflammatory, antioxidant, antithrombotic, hypoglycemic, antiseptic, and antibacterial properties [179,180]. Supplementation with lingonberry has been shown to prevent weight gain induced by a high-fat diet in animal models [181,182] and has demonstrated favorable effects on blood glucose, insulin levels, lipid profiles, and inflammatory markers. Mechanistically, lingonberry extract acts as a potent inhibitor of α-glucosidase and α-amylase activities, with reported IC50 values ranging between 12 and 17 μg/mL [183]. Clinical studies further support these findings; one trial revealed that the consumption of a sucrose-sweetened meal containing lingonberries significantly improved postprandial glycemic profiles compared to a similar meal without lingonberries [184]. In vitro studies have also shown that lingonberry extract can stimulate both basal and insulin-stimulated glucose uptake in skeletal muscle cells [185]. Bioactive compounds identified in lingonberries that may contribute to these metabolic effects include proanthocyanidins [186], quercetin [187], and resveratrol [188]. Additionally, the use of lingonberry press residue for extract production has shown promise. Purified polyphenol–polysaccharide conjugates derived from the press residue prevented weight gain in high-cholesterol-fed hamsters [189], while lingonberry pomace extracts demonstrated hypoglycemic effects in vitro through the inhibition of α-amylase and α-glucosidase activities [190]. Furthermore, supplementation with lingonberry skin extract was found to prevent increases in fasting blood glucose, body weight, and visceral fat accumulation in a mouse model of high-fat diet-induced obesity [191].

### 5.5. Bearberry (Arctostaphylos uva-ursi L.)

Bearberry (*Arctostaphylos uva-ursi* L.) is a perennial plant belonging to the *Ericaceae* (heather) family. Its leaves are particularly rich in arbutin, the primary bioactive compound, alongside other phytochemicals such as phenolic acids, flavonoids, and saponins [192]. Traditionally, aqueous infusions of bearberry leaves have been used for the treatment of various ailments. Due to its ecological vulnerability, bearberry is considered an endangered and protected species in several European countries [193]. Bearberry leaves (BLs) have long held a prominent place in folk medicine, valued for their abundance of secondary metabolites with important medicinal and pharmacological properties. The chemical composition of BL includes a diverse array of bioactive compounds such as gallic acid, ursolic acid, tannic acid, p-coumaric acid, galloylarbutin, gallotannins, quercetin, kaempferol, penta-O-galloyl-α-D-glucose, corilagin, picein, and hyperoside, among others [194]. Extracts from *Uvae ursi folium* have been employed as natural remedies for various conditions, including diuresis [195], and more recently as antioxidant agents in food packaging and skin-whitening agents in dermatological formulations [196]. Arbutin, the principal active compound (Figure 2), is particularly noted for its skin-depigmenting effects, exerting potent antimelanogenic and antioxidant activities [197].

Dried leaves of bearberry showed the potential to lower the risk of diabetic complications with the chemical composition found being the following: hydroquinone derivatives, arbutin, methylarbutica, and gallic acid in studies even older than 50 years [198,199]. Recent in vivo experimental studies have investigated the antidiabetic potential of extracts derived from bearberry leaves under conditions of experimentally induced insulin resistance (IR). Following two weeks of administration, the extracts demonstrated a significant capacity to lower blood glucose levels in rats, attenuate the progression of IR, and improve glucose tolerance. The observed hypoglycemic effect was comparable to that of metformin and was notably superior to the activity of Arphazetin [200]. Additional experimental evidence supports these findings, showing that bearberry leaf extracts effectively reduced blood glucose levels both in animals with induced pathology and in healthy animals subjected to glucose overload [201,202]. Furthermore, another study revealed that a dry alcoholic extract of bearberry leaves enriched with cysteine (PE50_cys) exerted notable hypoglycemic and pancreatic protective effects in a dexamethasone-induced IR model. Treatment with PE50_cys improved hyperglycemia and insulin resistance, and preserved β-cell mass which was otherwise reduced by dexamethasone exposure [203]. Moreover, a dry extract of bearberry leaves enriched with arginine exhibited hepatoprotective effects in diabetic rats. This extract enhanced the parameters of carbohydrate metabolism, including increased glycogen content in the liver and reduced blood lactate levels. The hepatoprotective effect is believed to be mediated primarily by the antioxidant properties of the plant-derived polyphenols within the PE50_arg composition, while the addition of arginine further amplified these beneficial outcomes [204].

### 5.6. Arbutus unedo, (The Strawberry Tree)

*Arbutus unedo (A. unedo),* commonly known as the strawberry tree, is a member of the *Ericaceae* family and is widely distributed throughout the Mediterranean region. It is also found in the Canary Islands and parts of western Asia, where the climatic conditions are favorable for its growth [205]. Traditionally, various parts of *A. unedo*, including its fruits, leaves, and roots, have been extensively utilized in folk medicine for their diuretic, astringent, antidiarrheal, antiasthmatic, anti-inflammatory, antidiabetic, antihypertensive, and anti-rheumatic properties, as well as for the treatment of gastrointestinal and renal disorders [206,207,208]. A wide range of pharmacological activities has been attributed to *A. unedo*, including astringent, depurative, anti-inflammatory, hemostatic, antitumor, antioxidant, antimicrobial, spasmolytic, and neuroprotective effects [209,210]. Experimental studies further confirm that extracts from *A. unedo* exhibit significant biological activities, demonstrating antioxidant, platelet antiaggregant, vasorelaxant, antihypertensive, and antidiabetic properties [211,212]. These diverse therapeutic effects are largely attributed to the plant’s rich phytochemical composition, which includes flavonoids, tannins, phenolic acids, organic acids, α-tocopherol, carotenoids, anthocyanins, triterpenoids, fatty acids, sterols, vitamin C, dietary fibers, and essential minerals such as calcium (Ca), potassium (K), magnesium (Mg), and phosphorus (P) [213,214,215]. Collectively, these bioactive compounds contribute to the remarkable pharmacological and nutritional profile of *A. unedo*.

**Figure 3:**
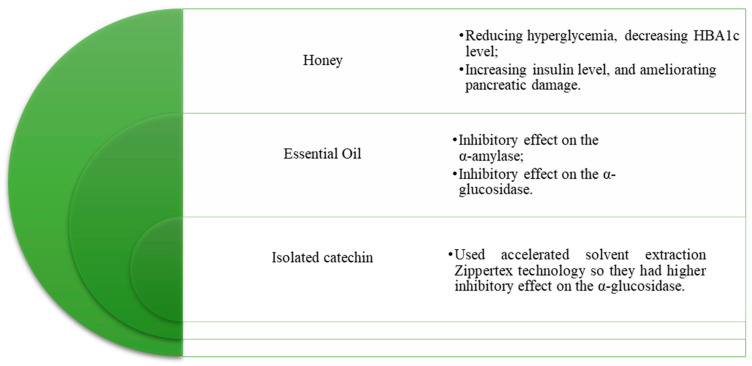
Therapeutic potential of Arbutus unedo products and isolates in type 2 diabetes mellitus (T2DM). The table highlights various A. unedo-derived products, including honey, essential oil, and isolated catechins, and their reported antidiabetic effects such as enzyme inhibition, glycemic control, increased insulin levels, and pancreatic protection [217,219,220].

In in vivo studies (Table 6), extracts of *Arbutus unedo* demonstrated a significant reduction in intestinal glucose absorption, which may partially explain the observed decrease in glycemia in the oral glucose tolerance test (OGTT) model [216]. The chronic oral administration of *A. unedo* extract over a four-week period in streptozotocin–nicotinamide (STZ–NA)-induced diabetic mice resulted in a marked decrease in blood glucose levels, comparable to the effects achieved with metformin, the positive control [217]. Furthermore, treatment with A. unedo extract was associated with the restoration of the histological architecture of the islets of Langerhans, suggesting a protective effect on pancreatic tissue in diabetic mice [217]. The hypoglycemic action of *A. unedo* is thought to involve the stimulation of insulin secretion from existing pancreatic β-cells or the mobilization of insulin from its bound forms [185,217]. Additionally, the chronic administration of the extract for four weeks significantly improved oral glucose tolerance and promoted weight reduction in rat models [218]. Beyond the roots, several studies have investigated other products derived from *A. unedo* in the context of T2DM management, as illustrated in Figure 3.

### 5.7. Crowberry

*Crowberry* (*Empetrum nigrum* L.) is a small genus of dwarf evergreen shrubs, recognized as a wild berry with significant potential for use in herbal medicine, largely due to its rich and diverse phenolic content [221]. Among its phytoconstituents, flavonols and benzoic acid derivatives are the most abundant soluble phenolic compounds identified in crowberry leaves [222]. In the context of diabetes management, several studies have explored the inhibitory effects of crowberry extracts on carbohydrate-digesting enzymes. Notably, extracts from the aerial parts of crowberry demonstrated potent α-glucosidase inhibitory activity, suggesting their potential to attenuate postprandial hyperglycemia by delaying carbohydrate digestion [223]. The antidiabetic activities of crude 70% ethyl alcohol extract and its fractions were analyzed to understand the biological activity of crowberry [224]. It was indicated that the significant inhibition of α-glucosidase and α-amylase activities by the ethyl acetate fraction (versus the other fractions) is due to the presence of polyphenolic compounds. Another study of solid phase extraction (SPE) on crowberry tried to enhance glucose uptake in liver cells. Among others, crowberries showed high stimulation of glucose uptake, which can lower blood glucose levels [225]. In a human study, Torronen et al. [226] investigated the fortification of blackcurrant juice with black crowberry powdered fruit extract and assessed its effects on polyphenol composition, urinary and plasma phenolic metabolites, and postprandial glycemic response in healthy subjects. Fortification doubled the TPC of the juice, increasing from 159 to 293 mg/100 mL. Following consumption, the urinary levels of metabolites such as dihydroxybenzoic acid sulfate and dihydroxyphenylacetic acid sulfate were significantly elevated, particularly after the intake of the fortified juice. Importantly, the combination of crowberry and blackcurrant improved the postprandial glycemic response following a 36 g sugar load, likely due to enhanced polyphenol bioavailability [133]. The ethanolic extract of the aerial parts of black crowberry demonstrated strong α-glucosidase inhibitory activity, reinforcing the potential of crowberry as a natural therapeutic agent to manage postprandial hyperglycemia and suggesting its possible use as an alternative antidiabetic treatment (Table 7) [203].

## 6. Toxicity and Safety Considerations

*Ericaceae*-derived phytochemicals, while promising for antidiabetic therapy, can exhibit notable toxicity at higher doses due to certain secondary metabolites. Grayanane diterpenoids (grayanoids) from genera like *Rhododendron* are a prime example—these diterpenes are notorious neurotoxins that hyperactivate voltage-gated sodium channels, leading to continuous nerve and muscle excitation [229]. Even slight overexposure can trigger acute neurocardiac symptoms: human “mad honey” poisoning cases from rhododendron nectar report hypotension, bradyarrhythmia, confusion, convulsions, and even atrioventricular block. Such effects reflect a narrow therapeutic index; beneficial doses can rapidly turn harmful as the concentration rises. Indeed, an EFSA risk assessment in 2023 found measurable cardiac toxicity in rats at ~15 µg/kg of grayanotoxin and warned that only very low levels (<0.05 mg/kg in honey) are considered safe to avoid acute intoxication. This dose-dependent hazard underscores the need for careful control of graminoid-containing preparations [230].

Phenolic glycosides like arbutin (abundant in *Arctostaphylos uva-ursi* and other *Ericaceae*) also present safety considerations. Arbutin itself is relatively benign, but it can hydrolyze to release hydroquinone—a compound with known genotoxic, carcinogenic, and organ-toxic effects upon prolonged exposure. High or chronic intake of arbutin-rich extracts could, thus, pose risks of liver and kidney damage if sufficient hydroquinone accumulates over time. In vitro studies support this mechanism: bearberry (*uva ursi*) extracts showed little cytotoxicity in bladder cell cultures until arbutin was fully converted to free hydroquinone, at which point marked cell toxicity was observed. Conversely, in vivo evidence indicates that toxicity is minimal at proper doses (a recent 90-day study in mice found that *Arbutus unedo* leaf extract (rich in arbutin and phenolics) caused no adverse effects up to 5000 mg/kg, aside from its desired hypoglycemic action). Notably, the estimated hydroquinone release at standard human doses of bearberry is about 11 µg/kg/day, far below the 100 µg/kg threshold for negligible risk. These findings highlight that appropriate dosing can keep arbutin’s metabolite well within safe margins [231].

Tannins, another common constituent in *Ericaceae* remedies, exemplify how dose determines remedy versus toxin. At moderate levels, tannins contribute antioxidant and antidiabetic benefits (e.g., slowing glucose absorption), but excessive intake can irritate the gastrointestinal tract and reduce nutrient bioavailability. Users of tannin-rich leaf teas occasionally report nausea or vomiting, an effect attributed to tannins’ astringent action on the gut mucosa. Prolonged high consumption may lead to liver stress—for instance, the chronic dosing of tannic acid in animals has produced hepatic injury. Such observations align with historical reports that sustained exposure to high tannin levels causes organ damage, although typical dietary or medicinal use is far lower. Therefore, proper preparation (e.g., decoction techniques that limit tannin concentration) and adherence to recommended dosages are crucial to mitigate these adverse effects [232].

Overall, the safety profile of *Ericaceae*-derived compounds is favorable when traditional usage guidelines are followed, but toxicological vigilance is warranted. Many potentially harmful constituents are present in only trace amounts in consumed preparations, and traditional processing often aims to reduce toxin content. Modern studies reinforce the importance of these practices: for example, carefully prepared *Rhododendron* extracts can exclude dangerous grayanoids, and refining *uva ursi* formulations can minimize free hydroquinone release. Future research should prioritize comprehensive toxicity assessments and risk evaluations for *Ericaceae* phenolics. Key gaps include the lack of chronic toxicity data (noted by regulators for compounds like grayanotoxin) and incomplete understanding of toxicological mechanisms (such as the molecular basis of grayanotoxin-induced genotoxicity). Addressing these gaps through long-term in vivo studies and mechanistic assays will help define clear safety margins. In addition, developing standardized extraction and dosing protocols can ensure that antidiabetic efficacy is achieved without accompanying harm. Such proactive safety profiling and dose optimization will be essential as *Ericaceae* phenolics move from traditional use to evidence-based clinical applications.

## 7. Perspective

*Ericaceae* family plants—including berry-bearing shrubs like *Vaccinium* (blueberries, cranberries, lingonberries) and *Gaultheria* (salal, wintergreen), as well as medicinal species of *Rhododendron* and *Arbutus* (strawberry tree)—have attracted interest for their antidiabetic potential. These plants are rich in polyphenolic compounds, especially flavonoids and pigmented anthocyanins, which confer potent antioxidant activity and contribute to hypoglycemic effects. The high antioxidant capacity of Ericaceous berries and leaves can mitigate diabetes-associated oxidative stress, while their bioactive constituents directly modulate carbohydrate metabolism. For instance, anthocyanin-rich *Vaccinium* extracts and related polyphenols can inhibit carbohydrate-digesting enzymes like α-glucosidase, slowing glucose absorption. Concurrently, flavonoids such as quercetin (abundant in *Vaccinium* spp.) have been shown to activate AMP-activated protein kinase and enhance glucose uptake in peripheral tissues, thereby improving insulin sensitivity. Similarly, *Arbutus unedo* yields catechin and other flavanols that act as strong α-glucosidase inhibitors, and *Rhododendron* flower extracts have demonstrated antihyperglycemic activity in diabetic models by promoting insulin-mediated glucose utilization. Collectively, the polyphenol-rich *Ericaceae* plants exhibit multifaceted antidiabetic mechanisms—from antioxidant protection to enzyme inhibition and improved glucose handling—underscoring their promise as functional foods or phytotherapeutic adjuvants for diabetes prevention and management.

## 8. Methodology

The search for information was conducted following the PRISMA (Preferred Reporting Items for Systematic Reviews and Meta-Analyses) guidelines, using Google Scholar, PubMed, and ScienceDirect. Articles published within the last 5 years were prioritized; however, older studies were included when more recent data were unavailable, provided they reflected the current state of research. The keywords used in the search included “*Ericaceae*”, in combination with “diabetes”, “diabetes mellitus”, “type 1 diabetes mellitus”, and “type 2 diabetes mellitus.” These terms were used primarily to gather general information on disease classification, complications, and pathophysiology. Additionally, “Vaccinium” (both generally and for each species individually) was combined with “diabetes”, and for each plant species discussed, both the Latin and common English names (e.g., “bilberry” and “crowberry”) were used to ensure comprehensive coverage. The inclusion/exclusion criteria emphasized studies addressing the core elements of diabetes mellitus, including its definition, classification, risk factors, complications, and pathophysiology, as well as those evaluating antidiabetic or hypoglycemic effects, specifically through α-amylase and α-glucosidase inhibition. After an initial search using Google Scholar, relevant data were verified and supplemented with full-text articles from PubMed and ScienceDirect, based on relevance to the review objectives.

## 9. Conclusions

The evidence gathered in this review highlights the significant antidiabetic potential of *Ericaceae* plants, primarily attributed to their rich content of phenolic compounds such as anthocyanins, flavonoids, and proanthocyanidins. These bioactive molecules exhibit multiple beneficial effects, including antioxidant, anti-inflammatory, and enzyme-inhibitory actions, contributing to improved glycemic control and insulin sensitivity. Both in vitro and in vivo studies confirm their capacity to modulate key metabolic pathways involved in diabetes pathophysiology. Given their efficacy, accessibility, and natural origin, the *Ericaceae* species represent a promising complementary strategy for diabetes prevention and management, warranting further clinical validation.

## Figures and Tables

**Table 1:** Flavonoids and anthocyanins in *Ericaceae* plants with antidiabetic effect, F—flavonoids; ANT—anthocyanins.

Species	Content Type	Identified Derivatives	Class
*V. myrtillus* (bilberry) 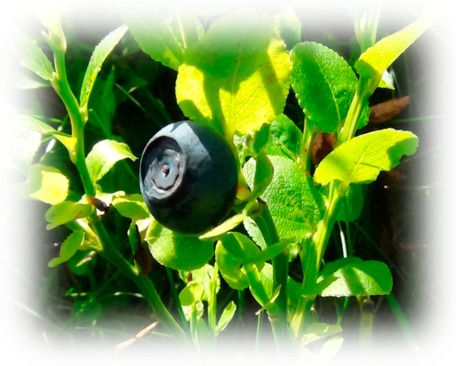	Cyanidin Delphinidin Malvidin Petunidin Myricetin Quercetin Others	cyanidin 3-galactoside, cyanidin 3-glucoside, cyanidin 3-arabinoside, peonidin 3-arabinoside, cyanidin 3-xyloside, cyanidin 5-glucoside, cyanidin 3,5-diglucoside delphinidin 3-galactoside, delphinidin 3-arabinoside, delphinidin 3-glucoside, malvidin 3-galactoside, malvidin 3-arabinoside, malvidin 3-glucoside petunidin 3-arabinoside, petunidin 3-acetylglucoside, petunidin 3-glucoside, myricetin 3-glucoside, myricetin 3-arabinoside, myricetin3-rhamnoside, myricetin-3-xyloside, myricetin 3-galactoside quercetin 3-arabinoside, quercetin 3-rhamnoside, quercetin 3-galactoside, quercetin 3-glucoside, quercetin 3-rutinoside, apigenin, chrysoeriol, isorhamnetin, laricitrin, syringetin, luteolin, kaempferol [54]	ANT ANT ANT ANT ANT F F
*V. macrocarpon* (cranberry) * 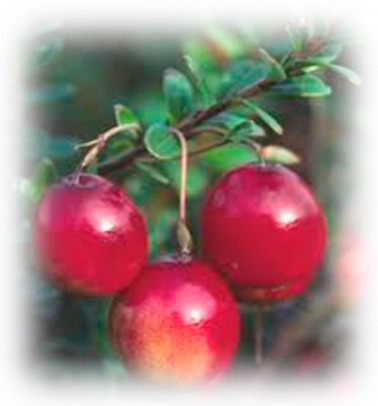 *	Cyanidin Delphinidin Malvidin Peonidin Pelargonidin Quercetin Kaempferol	cyanidin-3-glucoside, cyanidin-3-galactoside, cyanidin-3-arabinoside delphinidin-3-arabinoside malvidin-3-galactoside, malvidin-3-arabinoside peonidin-3-glucoside, peonidin-3-galactoside, peonidin-3-arabinoside pelargonidin-3-galactoside, pelargonidin-3-arabinoside quercetin-3-galactoside, quercetin-3-arabinoside, quercetin-3-rhamnoside kaempferol-3-glucoside [44]	ANT ANT ANT ANT ANT F F
*Vaccinium* spp. (blueberry) 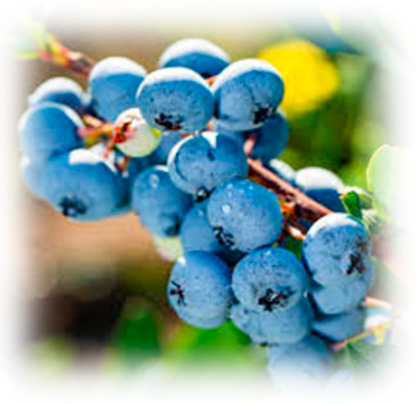	Cyanidin Delphinidin Malvidin Petunidin Myricetin Quercetin	cyanidin 3-galactoside, cyanidin 3-glucoside, cyanidin 3-arabinoside delphinidin 3-arabinoside, delphinidin 3-acetylglucoside malvidin 3-galactoside, malvidin 3-glucoside, malvidin 3-arabinoside, malvidin 3-acetylglucoside petunidin 3-galactoside, petunidin 3-glucoside, petunidin 3-arabinoside myricetin 3-galactoside, myricetin 3-glucoside, myricetin 3-rhamnoside quercetin 3-galactoside, quercetin 3-glucoside, quercetin 3-glucosylxyloside, quercetin 3-rutinoside, quercetin 3-xyloside [55]	ANT ANT ANT ANT F F
*V. vitis* idaea (lingonberry) 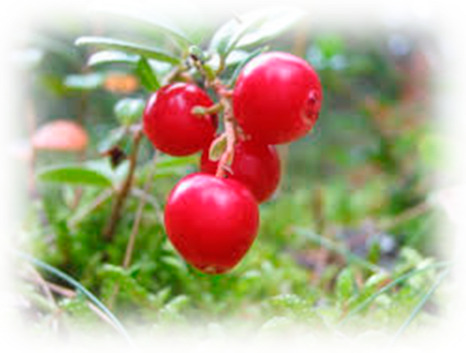	Cyanidin Delphinidin Petunidin Peonidin Myricetin Quercetin Kaempferol Isorhamnetin	cyanidin 3-glucoside, cyanidin 3-arabinoside delphinidin 3-glucoside, delphinidin 3-arabinoside, delphinidin 3-galactoside, petunidin 3-galactoside, petunidin 2-glucoside, peonidin 3-arabinoside, peonidin 3-glucoside, peonidin 3-galactoside myricetin 3-glucoside quercetin 3-glucoside, quercetin 3-galactoside, quercetin 3-arabinoside, quercetin 3-xyloside kaempferol 3-rhamnoside, kaempferol 3-glucoside isorhamnetin 3-galactoside, isorhamnetin 3-glucoside [56]	ANT ANT ANT ANT F F F F
*Arctostaphylos uva-ursi* L. (Bearberry) 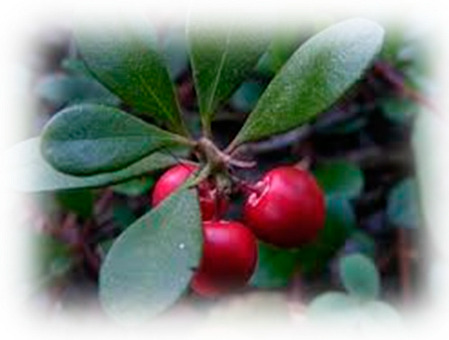	Quercetin Kaempferol Myricetin	Isoquercetin, quercitin-3-gentiobioside, hyperoside, avicularin, rutin Kaempferol-pentoside/hexoside, Myricetin-pentoside/hexoside [57]	F F F
*Vaccinium arctostaphylos* (Caucasian whortleberry) 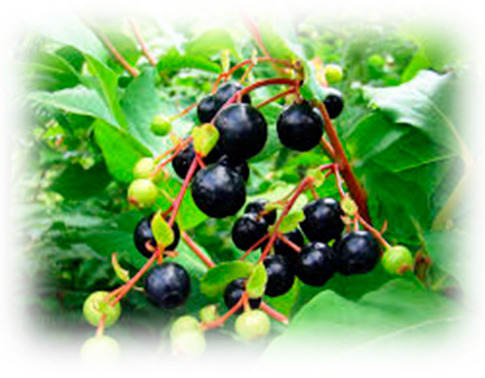	Delphinidin Petunidin Malvidin Cyanidin	delphinidin 3-galactoside, delphinidin 3-arabinoside, delphinidin 3-glucoside petunidin 3-arabinoside, petunidin 3-glucosidemalvidin 3-galactoside, malvidin 3-arabinoside, malvidin 3-glucoside cyanidin-3-O-xyloside [58]	ANT ANT ANT ANT
*Gaultheria trichophylla (Himalayan snowberry)* 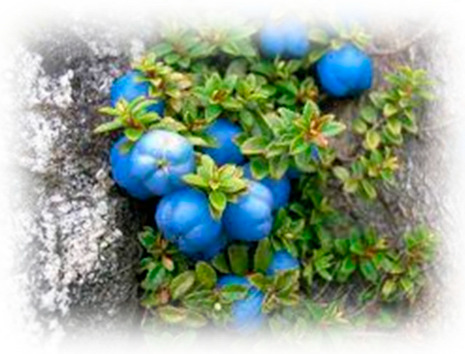	Quercetin Kaempferol Catechin Epicatechin	Quercetin 3- glycoside, Quercetin 3-O-galactoside, Quercetin 3-rhamnoside, Kaempferol 7- glucoside, Kaempferol 3-glucoside [59]	F
*Rhododendron arboreum* (Himalayan rhododendron) 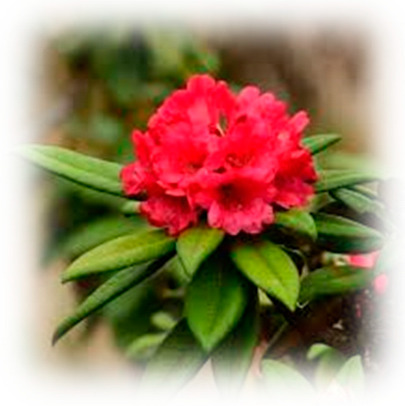	Ursolic acid derivatives Quercetin	quercetin-3-O-galactoside [60]	F
*Rhododendron groenlandicum* (Labrador tea) 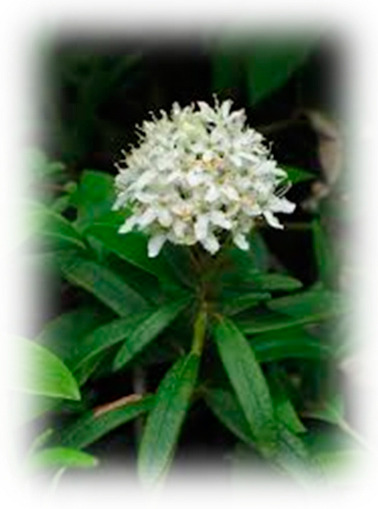	Catechin Epicatechin [61]		F
*Arbutus unedo* 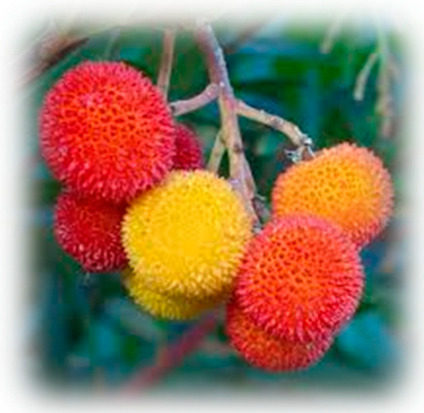	Myricetin Quercetin Kaempferol	Myricetin 3-glucoside, Myricetin 3-pentosideQuercetin 3- glycoside, Quercetin 3-O-galactoside, Quercetin 3-rhamnoside, Kaempferol 7- glucoside, Kaempferol 3-glucoside [62]	F F F
*Empetrum nigrum* (Crowberry) 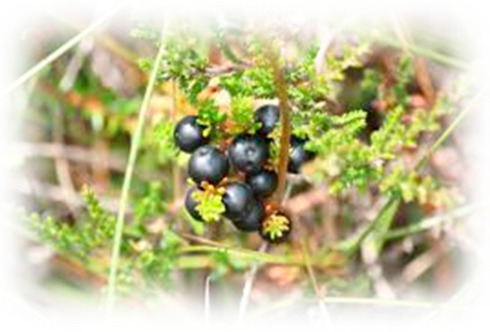	Cyanidin Delphinidin Petunidin Malvidin Quercetin Kaempferol	cyanidin 3-galactoside, cyanidin 3-glucoside, cyanidin 3-arabinoside delphinidin 3-galactoside, delphinidin 3-arabinoside, delphinidin 3-glucoside petunidin 3-arabinoside, petunidin 3-glucosidemalvidin 3-galactoside, malvidin 3-arabinoside, malvidin 3-glucoside quercetin-3-glucoside, quercetin-3-arabinose, quercetin-3-xyloside, quercetin-3-galactoside, quercetin-3-rhamnoside glucoside and galactoside forms [63]	ANT ANT ANT ANT F F

**Table 2:** Chemical structure of the main flavonoids and anthocyanins from *Ericaceae*.

Compound	IUPAC Name	Molecular Formula	2D Structure
Quercetin [64]	2-(3,4-dihydroxyphenyl)-3,5,7-trihydroxychromen-4-one	C_15_H_10_O_7_	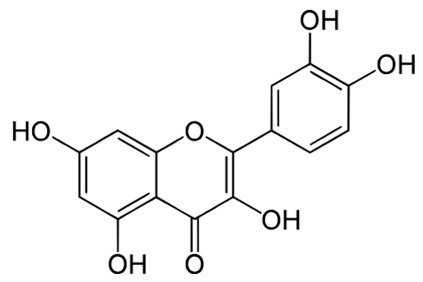
Kaempferol [65]	3,5,7-trihydroxy-2-(4-hydroxyphenyl)chromen-4-one	C_15_H_10_O_6_	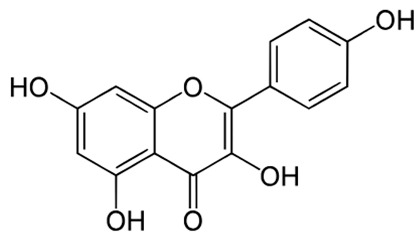
Myricetin [66]	3,5,7-trihydroxy-2-(3,4,5-trihydroxyphenyl)chromen-4-one	C_15_H_10_O_8_	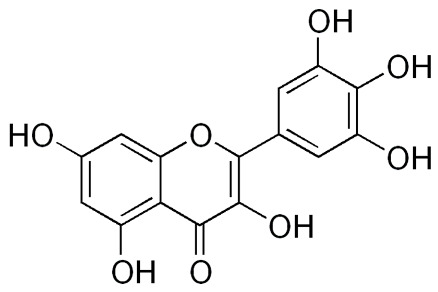
Cyanidin [67]	2-(3,4-dihydroxyphenyl)chromenylium-3,5,7-triol	C_15_H_11_O_6_	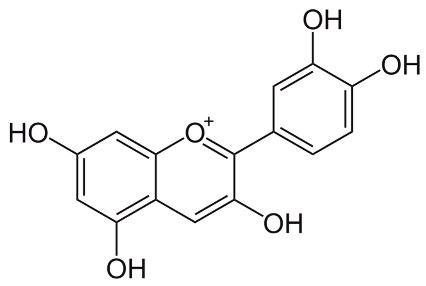
Delphinidin [68]	2-(3,4,5-trihydroxyphenyl)chromenylium-3,5,7-triol;	C_15_H_11_O_7_	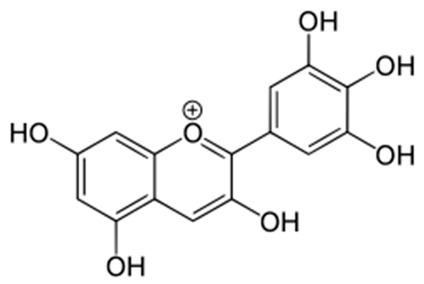
Malvidin [69]	(2*S*,3*R*,4*S*,5*S*,6*R*)-2-[5,7-dihydroxy-2-(4-hydroxy-3,5-dimethoxyphenyl)chromenylium-3-yl]oxy-6-(hydroxymethyl)oxane-3,4,5-triol	C_23_H_25_O_12_	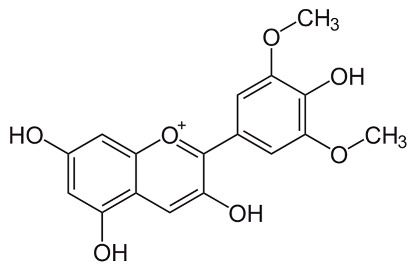
Petunidin [70]	C_22_H_23_O_12_	(2*S*,3*R*,4*S*,5*S*,6*R*)-2-[2-(3,4-dihydroxy-5-methoxyphenyl)-5,7-dihydroxychromenylium-3-yl]oxy-6-(hydroxymethyl)oxane-3,4,5-triol	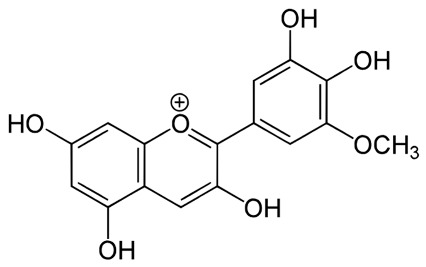
Pelargonidin [71]	C_15_H_11_ClO_5_	2-(4-hydroxyphenyl)chromenylium-3,5,7-triol;chloride	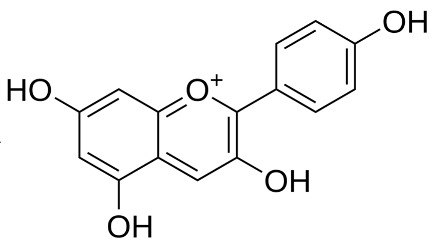

**Table 3:** Total phenolic content of selected *Ericaceae* plants (mg GAE/100 g FW or g DW) GAE = Gallic Acid Equivalents; FW—fresh weight; DW—dry weight.

Species	Variety	Total Phenolic Content	Reference
*V. myrtillus*	Wild bilberry	492–563 mg GAE/100 g FW	[72]
*V. macrocarpon*	“Early Black” cultivated	~441 mg GAE/100 g FW	[73]
*Vaccinium* spp.	“Bluecrop” highbush blueberry	~327 mg GAE/100 g FW	[72]
*V. vitis-idaea*	Wild lingonberry	468–661 mg GAE/100 g FW	[74]
*Arctostaphylos uva-ursi* L.	Leaf extracts (wild populations)	238.85–318.28 mg GAE/g DW	[75]
*Vaccinium arctostaphylos*	Wild fruit (Caucasian whortleberry)	389–578 mg GAE/100 g FW	[76]
*Gaultheria trichophylla*	Wild fruit (Himalayan snowberry)	~3.71 mg GAE/g FW (≈371 mg/100 g FW)	[77]
*Rhododendron arboreum*	Flower petals (Himalayan rhododendron)	~4.89 mg GAE/g DW (best processing)	[78]
*Rhododendron groenlandicum*	Leaves (Labrador tea)	20 g/100 g DW (leaf extract)	[79]
*Arbutus unedo*	Strawberry tree fruit	16.78–25.86 mg GAE/g DW	[80]
*Empetrum nigrum*	Wild black crowberry (Canada)	~454 mg GAE/100 g FW (fresh fruit)	[75]

**Table 4:** Antioxidant activity of total phenolic compounds from *Ericaceae* Plants.

Species	Main Compounds Class Responsible for Antioxidant Activity	ABTS	DPPH
*V. myrtillus*	Anthocyanins (anthocyanidin glycosides)—e.g., delphinidin and cyanidin derivatives	60.9–106.0 µmol Trolox/g FW (ABTS radical cation scavenging) [81]	216.5–376.8 µmol Trolox/g FW (DPPH radical scavenging [82]
*V. macrocarpon*	Proanthocyanidins (A-type PAC oligomers)	189–264 µmol Trolox/g DW (ABTS, cultivar range) [83]	214–320 µmol Trolox/g DW (DPPH, cultivar range) [84]
*Vaccinium* spp.	Anthocyanins (flavonoid pigments)—malvidin, petunidin, and cyanidin glycosides	~259.9 µmol Trolox/g DW (ABTS in highbush cv. ‘Biloxi’) [85]	~214.1 µmol Trolox/g DW (DPPH in ‘Biloxi’ fruit) [85]
*V. vitis idaea*	Anthocyanins (cyanidin glycosides)	~74.3–104.0 µmol Trolox/g DW (TEAC/ABTS assay) [80]	Potent DPPH scavenging capacity (multiple radicals quenched; ORAC/DPPH assays confirm high activity) [86]
*Arctostaphylos uva-ursi* L.	Phenolic glycosides (arbutin)	173.5–643.7 mg Trolox/g (ABTS•⁺ scavenging, water vs. EtOH extracts) [87]	377.4–821.8 mg Trolox/g (DPPH scavenging, water vs. EtOH) [87]
*Vaccinium arctostaphylos*	Anthocyanins (delphinidin, petunidin, and malvidin glycosides)	~19.5 µmol Trolox/g FW [76]	SC50 = 0.14 mg/mL (ethanol extract) [88,89]
*Gaultheria trichophylla*	Polyphenols (flavonoids and tannins)	4.35 mM AAE/100 g FW [77]	2.56 mM AAE/100 g FW [77]
*Rhododendron arboreum*	Anthocyanins and flavonoids (quercetin derivatives)	21.25–31.87 mM AAE/100 g DW [78]	22.59–36.61 mM AAE/100 g DW [78]
*Rhododendron groenlandicum*	Flavonol glycosides, catechins (quercetin, myricetin, and catechin)	-	Strong radical scavenging activity (ORAC assay confirms high potency) [90]
*Arbutus unedo*	Flavan-3-ols (catechin and gallocatechin tannins)	74.3–104.0 µmol Trolox/g DW (antioxidant capacity, TEAC assay) [80]	DPPH radical scavenging EC50 in low mg/mL; activity strongly correlates with total phenolics [80]
*Empetrum nigrum*	Anthocyanins	107 µmol Trolox/g [91]	~90 mg Trolox/g [89,91]

**Table 5:** Studies on *Vaccinium* species on diabetes mellitus.

Plant	Age	Number	Duration	Results	Reference
*Blueberry*	27 ± 5	34	18 weeks	Lower C-reactive protein and blood glucose levels compared to the control group	[111]
	22–65	17	/	Significant increase in pancreatic polypeptide(PP) concentrations in intervention group	[112]
	51–75	58	8 weeks	Lower hemoglobin A1c, fructosamine, and triglycerides	[113]
*Bilberry*	30–65	105	90 days	Reduced 2 h blood postprandial glucose and homeostasis model assessment of insulin resistance (HOMA-IR) scores	[114]
	25–60	47	8 weeks	Significant increase in fasting serum hippuric acid in intervention group	[115]
	55.8 ± 9.5	20	4 weeks	Tendency of improved glycemic control in intervention group	[116]
*Cranberry*	40–75	160	12 weeks	Reduced HbA1c, low-density lipoprotein-c, apolipoprotein A-1, apolipoprotein B in intervention group	[117]
	56–67	58	24 weeks	Decreased serum LDL cholesterol, triglycerides, apolipoprotein B, and apo C-III; increased HDL cholesterol	[118]
	56 ± 6	25	/	Lower postprandial increases in glucose at 2 and 4 h in the cranberry group,	[119]
	25–65	56	8 weeks	Reduced circulating TGs, CRP, glucose, insulin resistance, and diastolic BP in intervention group	[120]
*Llingonberry*	25–69	20	/	Improved postprandial glycemic profiles	[74]

**Table 6:** In vivo studies on *A. unedo* roots in T2DM.

Plant Part	Extract/Fraction	Model Applied	Effect/Mechanism of Action	Reference
Roots	Water	OGTT * IVGTT *	Antihyperglycemic effect Inhibition of jejunal glucose absorption	[216]
Roots	Water	OGTT n-str-induced diabetic rats	Hypoglycemic effect Potentiation of the insulin Activity Improved glucose peripheral consumption	[212]
Roots	Catechin	α-glucosidase assay	Antidiabetic effect through inhibition of α-glucosidase enzyme	[213]
Roots	Water	α–glucosidase and α-amylase assays	Regeneration of pancreatic C-cells	[217]
Roots bark	Water	OGTT	Improved oral glucose tolerance	[218]

* OGTT—oral glucose tolerance test; IVGTT—intravenous glucose tolerance test.

**Table 7:** *Ericaceae* species with in vitro antidiabetic activity and potential for in vivo efficacy.

Species	Experimental Model	Observed Activity	Main Compounds
Gaultheria hispidula (Creeping snowberry) [185]	C2C12 myotubes and 3T3-L1 adipocytes (cell assays)	Stimulated glucose uptake; promoted adipogenesis (~50% of rosiglitazone); antioxidant and neuroprotective activity	Phenolic glycosides (e.g., gaultherin and arbutin) and flavonoids
Rhododendron tomentosum (Marsh Labrador tea) [227]	C2C12, 3T3-L1 adipocytes, and antioxidant assays	Strong glucose uptake stimulation; PPARγ-like adipogenic activity; potent antioxidant; no insulin secretion	Flavonoids, catechins, and polyphenols
Gaultheria shallon (Salal berry) [228]	α-amylase, α-glucosidase, and DPP-IV enzyme assays	Inhibited α-glucosidase, α-amylase (>60%), DPP-IV (~56%); high antioxidant potential	Anthocyanins, procyanidins, and flavonoids

## Data Availability

Data are contained within the article.
